# Design and Evaluation of an Intensive Care Unit Dashboard Built in Response to the COVID-19 Pandemic: Semistructured Interview Study

**DOI:** 10.2196/49438

**Published:** 2023-09-26

**Authors:** Marceli Wac, Ian Craddock, Sofia Chantziara, Tabitha Campbell, Raul Santos-Rodriguez, Brittany Davidson, Chris McWilliams

**Affiliations:** 1 Faculty of Engineering University of Bristol Bristol United Kingdom; 2 University Hospitals Bristol and Weston NHS Foundation Trust Bristol United Kingdom

**Keywords:** software engineering, dashboard, interactive display, COVID-19, intensive care, critical care, intensive care unit, ICU, human-centered design, participatory design, health, design, interview, electronic health record, EHR, electronic patient record, EPR, clinical information system, CIS, thematic analysis

## Abstract

**Background:**

Dashboards and interactive displays are becoming increasingly prevalent in most health care settings and have the potential to streamline access to information, consolidate disparate data sources and deliver new insights. Our research focuses on intensive care units (ICUs) which are heavily instrumented, critical care environments that generate vast amounts of data and frequently require individualized support for each patient. Consequently, clinicians experience a high cognitive load, which can translate to suboptimal performance. The global COVID-19 pandemic exacerbated this problem by generating a large number of additional hospitalizations, which necessitated a new tool that would help manage ICUs’ census. In a previous study, we interviewed clinicians at the University Hospitals Bristol and Weston National Health Service Foundation Trust to capture the requirements for bespoke dashboards that would alleviate this problem.

**Objective:**

This study aims to design, implement, and evaluate an ICU dashboard to allow for monitoring of the high volume of patients in need of critical care, particularly tailored to high-demand situations, such as those seen during the COVID-19 pandemic.

**Methods:**

Building upon the previously gathered requirements, we developed a dashboard, integrated it within the ICU of a National Health Service trust, and allowed all staff to access our tool. For evaluation purposes, participants were recruited and interviewed following a 25-day period during which they were able to use the dashboard clinically. The semistructured interviews followed a topic guide aimed at capturing the usability of the dashboard, supplemented with additional questions asked post hoc to probe themes established during the interview. Interview transcripts were analyzed using a thematic analysis framework that combined inductive and deductive approaches and integrated the Technology Acceptance Model.

**Results:**

A total of 10 participants with 4 different roles in the ICU (6 consultants, 2 junior doctors, 1 nurse, and 1 advanced clinical practitioner) participated in the interviews. Our analysis generated 4 key topics that prevailed across the data: our dashboard met the usability requirements of the participants and was found useful and intuitive; participants perceived that it impacted their delivery of patient care by improving the access to the information and better equipping them to do their job; the tool was used in a variety of ways and for different reasons and tasks; and there were barriers to integration of our dashboard into practice, including familiarity with existing systems, which stifled the adoption of our tool.

**Conclusions:**

Our findings show that the perceived utility of the dashboard had a positive impact on the clinicians’ workflows in the ICU. Improving access to information translated into more efficient patient care and transformed some of the existing processes. The introduction of our tool was met with positive reception, but its integration during the COVID-19 pandemic limited its adoption into practice.

## Introduction

### Background

Intensive care units (ICUs) are busy and complex environments in which patients require continuous monitoring and multiple organ support. The staff must be alert to many data sources such as vital signs and laboratory test results. The heterogeneous nature of the patients in the ICUs frequently necessitates an individualized approach from clinicians, resulting in as many as 200 interventions per patient per day [1]. This translates to a high cognitive load imposed on the staff. Information overload [2] and poor communication [3,4] have been found to negatively affect patient safety and outcomes. A system that can help clinical staff retrieve and process key information about a patient’s condition has the potential to benefit patient outcomes [4,5].

As data-driven, interactive, and visual tools, dashboards are used to consolidate and present data from multiple sources, help ascertain and monitor trends, and inform about the status of key indicators for a patient’s health condition [6-9]. Dashboards can help reduce cognitive load, promote data-driven decision-making, and improve adherence to evidence-based practice guidelines, resulting in improved patient outcomes [5-7,10]. The use of dashboards and interactive displays has been linked to more accurate and faster clinical care decision-making in the ICUs and critical care settings [11]. There is also evidence demonstrating the efficacy of using dashboards to improve patient care solely by promoting better access to relevant information [10].

The use of dashboards is becoming increasingly popular in health care services, as an increasing amount of patient information is digitized [12]. Research has highlighted a number of user requirements, including customizability (eg, adapting displayed data to parameters of interest for each of the staff members individually), dynamic presentation of data (eg, highlighting trends, detecting and representing change and urgency, and retrieving recent data), task management (eg, summarizing data for easier sharing with colleagues, recalling and tracking information for sharing, tracking tasks, and managing staff workloads), and organization of information based on medical concepts (eg, structuring information by organ systems, classifiers, and problems) [13-17].

The COVID-19 pandemic resulted in an increase in hospitalizations, especially in the early months, putting additional strain on ICU resources, specifically critical care beds with mechanical ventilation [18,19]. To cater to this influx of patients, the National Health Service (NHS) set out to rapidly build additional field hospitals called Nightingale Hospitals, which would accommodate the surplus admissions. In early 2020, the 2 local ICUs in University Hospitals Bristol and Weston NHS Foundation Trust (UHBW) in Bristol, United Kingdom, reported a critical need for an IT solution to help their staff manage the increased patient caseloads. The outline brief from the units envisaged a dashboard that would pull together disparate data sources in the ICU to help reduce the cognitive load on extremely busy clinical staff. A particular concern was that staff-to-patient ratios, and hence patient safety, would be eroded by a combination of massively increased patient numbers and COVID-19 cases among their trained staff. The guidelines for the provision of intensive care medicine suggest a ratio of 1 consultant to 10 patients [20]. These numbers were drastically reduced during the COVID-19 pandemic, allowing a consultant to look after up to 30 patients [21], and the NHS Nightingale Hospitals assumed a worst-case contingency ratio of 1 consultant to 60 patients. During the early stages of this research, it was envisioned that this new system would be deployed not only in the UHBW but also in the Bristol Nightingale Hospital, which would have been among the largest digital ICUs in the world, with an intensive care bed capacity of 300 [22].

To summarize, we conducted a qualitative study that focused on capturing the software requirements for a dashboard [23]. The study involved interviews with clinical staff, which were structured to elicit requirements for a bespoke dashboard that would allow for monitoring of the high volume of patients, particularly during the COVID-19 pandemic. We found that the ICU staff had the following requirements (R1-R5) for the dashboard:

1. *R1: Flexibility with changing protocols* for an evolving disease, where functionality can be updated quickly and effectively to respond to emerging information about the management of this new disease
2. *R2: A mobile dashboard* that staff would be able to use while attending to patients in the ICU
3. *R3: Customizability* allowing individual users to tailor the appearance of the dashboard to suit their role
4. *R4: Real-time analysis* delivered as data visualizations to help busy ICU staff understand patients’ clinical trajectories
5. *R5: Task and staff management* allowing to track both staff and patient movements, deliver handovers, and monitor tasks to ensure timely delivery of care.

### Objectives

This study describes the development and usability testing of an ICU dashboard that we built in response to the requirements R1-R5 and that could pull together disparate data sources in the ICU to help reduce the cognitive load on busy clinical staff and support their increased workload during the COVID-19 pandemic. The dashboard was developed based on the requirements captured during the interviews with the key stakeholders at UHBW [23]. Together with the capacity assumptions made with regard to deployment in the Nightingale Hospital Bristol, these requirements informed the core set of features of the dashboard, the software development life cycle, and the architecture of the system.

In this study, we aim to outline the design process of the new dashboard and evaluate its use in practice. Our goal was to understand the impact that the introduction of the dashboard had on patient care and the workflows of clinicians within the ICU. The design methodology is presented and contextualized within the study setting to establish how it informed the development of the dashboard and showcase the software we built. The evaluation focused on the relevance of the initial requirements gathered directly from the end users of the system, the barriers to effective deployment within the ICU, and the challenges of developing digital tools during the COVID-19 pandemic. By presenting our findings, we aim to highlight the key friction points in the deployment process and inform the future efforts of developing dashboards in the clinical setting.

## Methods

### Software Development Methodology

In addition to the 5 core requirements established during the interviews with the stakeholders from UHBW, several other key requirements were imposed on the dashboard software. First, owing to the circumstances in which the dashboard was being developed—the rapidly progressing global COVID-19 pandemic—the software was needed immediately and therefore had to have been built in a short span of time. Second, this constantly evolving situation required clinicians to adapt their ways of working as the official safety recommendations and treatment guidelines continued to change. This transformative nature of the requirements suggested the need for a robust framework that would allow the software development to move forward, adapting to the changing requirements with minimal delay to the dashboard delivery. To facilitate this, the development followed the Rapid Application Development methodology, focusing on iterative prototype development, rapid delivery, and frequent liaison with stakeholders [24]. The software was designed and implemented in <7 months (April 13 to November 8, 2020) by a team of 2 developers (a back-end developer [CM] responsible for integration with existing systems at the trust and development of the backing services and 1 full-stack developer [MW] responsible for the design and development of the front-end interface and backing services), who volunteered to work on the dashboard. The development cycle followed the *prototype-test-refine* loop and prioritized the delivery of the working product over the write-up of the research and sharing learnings with the participants.

### Capturing Requirements in Software Design

To facilitate the functional requirement of a mobile dashboard (R2) and the ability to adapt to an evolving care process (R1), the dashboard was developed in a form of an internally hosted progressive web application (PWA). This approach provided 2 key advantages over building a native application: it enabled access to the software from all types of devices regardless of the underlying operating system or the device type (eg, mobile phone, tablet, and desktop computer); it also enabled application updates to be automatically distributed to all client devices without requiring manual updates by individual users.

**Figure 1:**
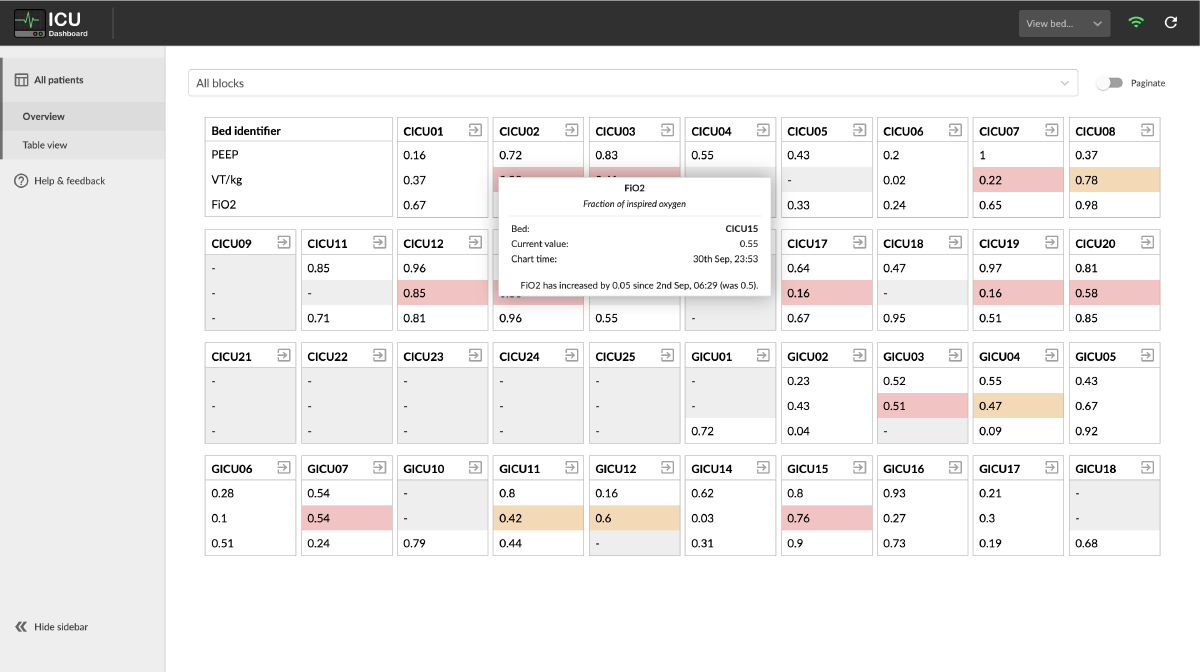
Ward overview allowed the participants to view a snapshot of the entire intensive care unit ward on a single screen in an interactive format. Focusing on the measurement would provide further information.

**Figure 2:**
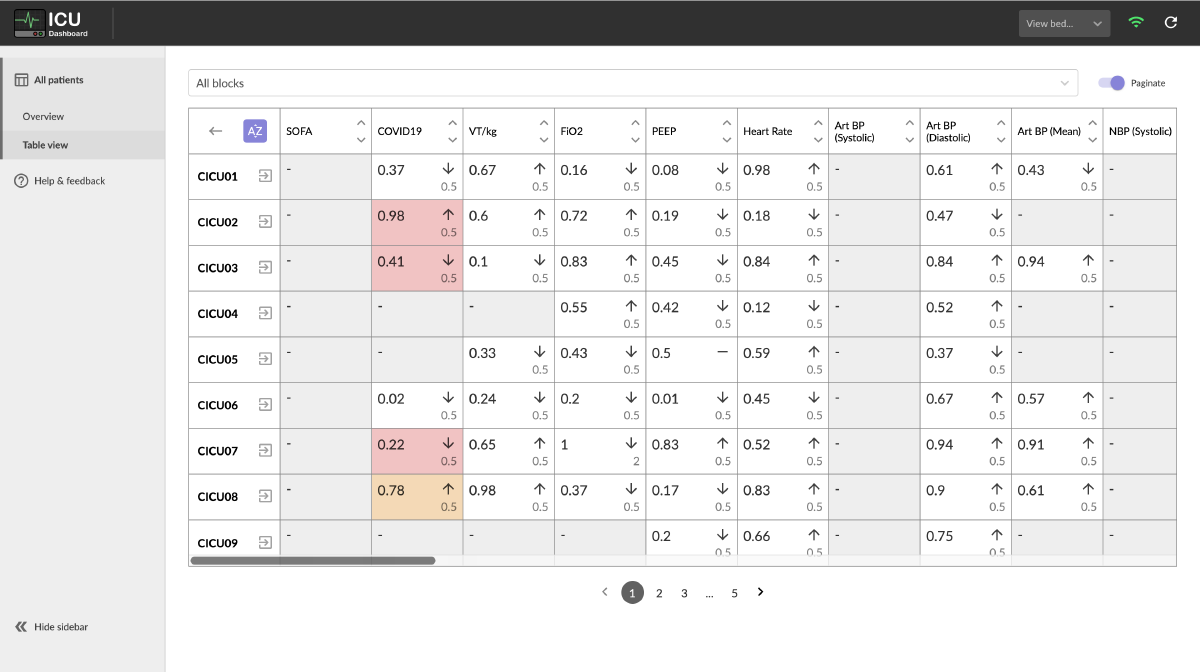
Table view displayed more data points per bed and provided trend indicators to suggest how the value has changed since the last measurement. Each cell contained the current and previous value as well as a trend indicator suggesting the temporal change; the colored highlight was used to call attention to values outside their predefined normal ranges.

**Figure 3:**
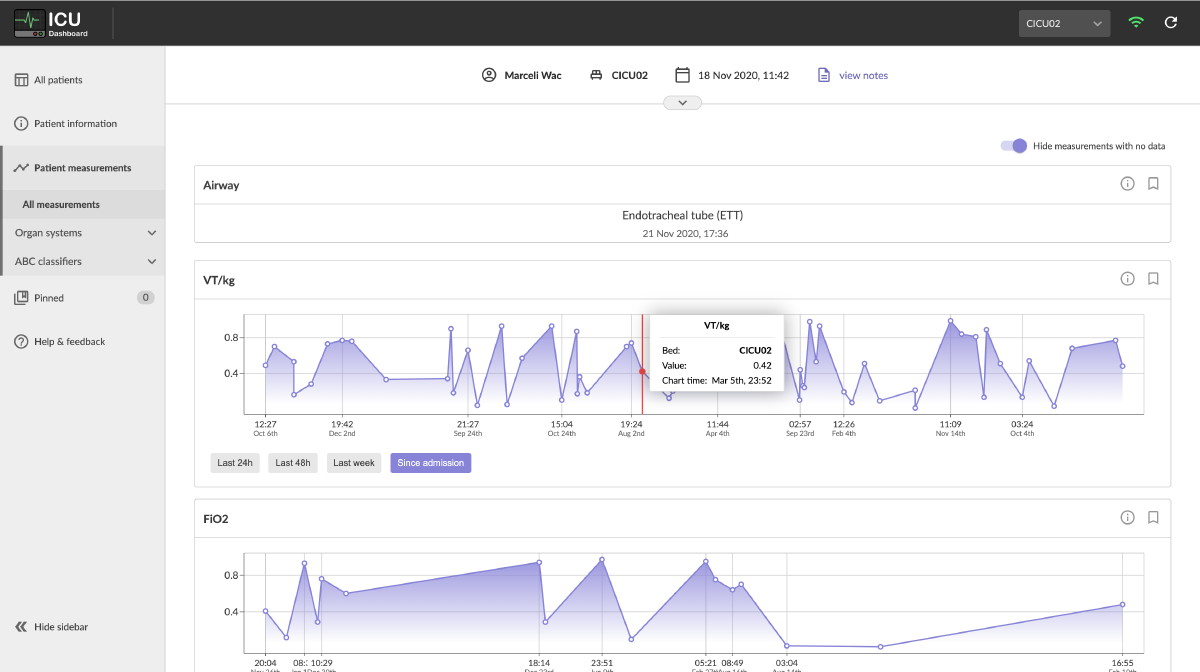
Bed view displayed the details of a single patient in a graphical and interactive format. Focusing on the chart would provide detailed information for the selected point in time.

To cater to a wide variety of roles involved in the delivery of care in the ICUs, the software incorporated multiple views of the data, allowing for granular control over the breadth and depth of the displayed information. This enabled each user to tailor the type and amount of information presented to them on the dashboard depending on their role or current task in an effort to satisfy the requirement of customizability (R3). To that extent, 3 subpages were developed: a ward overview presenting 3 key metrics for each of the currently occupied beds (Figure 1); a table view that displayed a matrix of parameters across the occupied beds (Figure 2); and a bed view that detailed the information for a single patient, including their demographics and free-form text notes, as well as time-series parameters that were visualized using line charts (Figure 3).

Although the parameters displayed in each view could be changed to cater to the changing needs of the stakeholders, the bed view facilitated further customization by allowing each user to filter displayed parameters based on organ systems or Airway, Breathing, Circulation, Disability, Exposure classifiers (as outlined in the study by Smith and Bowden [25]), pin-selected parameters and view them together, adjust the time range for the displayed charts, and automatically hide parameters without data points to display (Figure 3).

The data visualization and analytics requirement (R4) was addressed in both the table view’s trend indicators and value-based highlighting (Figure 2) as well as in the parameter charts drawn for each patient individually in bed view (Figure 3). Finally, because of the limitations of the software’s integration with existing systems, such as the lack of ability to authenticate users, the task management requirement (R5) was primarily addressed by providing aggregated information in the ward view, which aimed to facilitate handovers and other collaborative tasks.

### Participant Recruitment

The dashboard was deployed in the ICUs using servers already embedded in the UHBW’s infrastructure and made accessible to devices on the internal network. To maximize the potential benefits of easier access to the information, a training video showcasing the features of the dashboard was recorded and shared with all the staff located within the ICUs. After 25 days following the deployment, a recruitment email was sent to the ICU staff inviting them to participate in the interviews aimed at evaluating the usefulness and effectiveness of the dashboard. In total, 10 participants were recruited (of whom 6 were consultants, 2 were junior doctors, 1 was a nurse, and 1 was an advanced clinical practitioner) and interviewed over the course of 99 days to capture their impressions of the dashboard.

### Data Collection

We conducted 10 semistructured interviews that followed a topic guide that served as a baseline for an interviewer and aimed to encourage consistency between different participants (Multimedia Appendix 1). The questions in the topic guide focused on exploring the modes of use and general impressions of the dashboard. These questions were supplemented by additional questions asked post hoc, which further probed any potential themes established during the interview. Each interview lasted approximately 30 to 45 minutes and was recorded and transcribed for later analysis. Owing to the pandemic situation present at the time of the recording, all interviews were conducted exclusively remotely over the internet.

### Thematic Analysis

Various theories were previously developed to understand technology acceptance [26,27], which refers to the adoption and use of technologies for the tasks they were designed to support [28]. These theories have introduced several factors that can affect user acceptance and adoption of new technologies. According to the Technology Acceptance Model (TAM) [29], perceived usefulness and perceived ease of use can influence the uptake of technologies [30].

We combined both inductive and deductive approaches to thematic analysis to devise a thematic framework for evaluating the dashboard. The inductive aspect of our framework involved open coding, during which all the interviews were coded iteratively and followed the principles of thematic analysis, as outlined by Braun and Clarke [31]. Subsequently, we analyzed the interviews again, this time focusing on the driving factors of the TAM—the perceived usefulness and perceived ease of use, which were imposed as additional codes. The established codes were compared across the interviews and structured into themes, which were later discussed by the authors. The analysis was performed using NVivo 12 (Lumivero) [32].

### Ethical Considerations

This study was approved by the Faculty of Engineering Research Ethics Committee at the University of Bristol (case 2020-3236).

## Results

### Overview

The thematic analysis generated a total of 19 themes surrounding specific thoughts and opinions on the dashboard and participants’ experiences in the ward. These themes were then aggregated into topics and subtopics to establish a narrative structure for the purpose of disseminating the results. Crucially, neither topics nor subtopics held any coded data themselves.

### Topic: the Dashboard Met the Usability Expectations of the Participants

#### Overview

**Figure 4:**
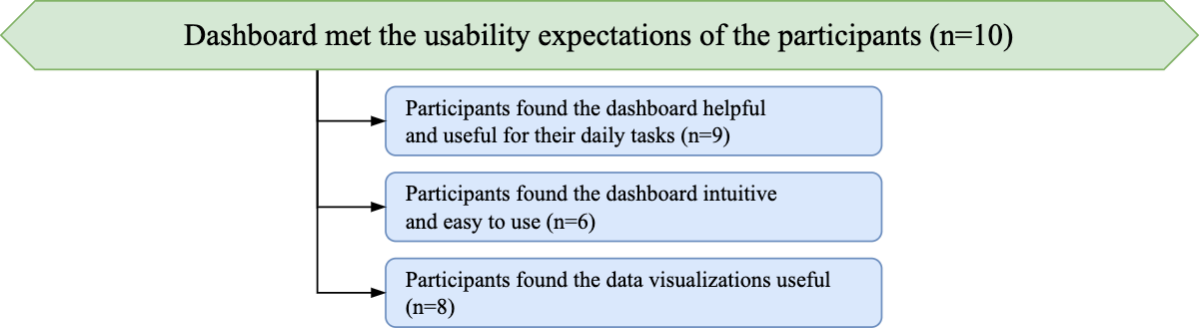
Hierarchy of the theme structure for the topic “The dashboard met the usability expectations of the participants.”.

Following the introduction of the dashboard, all 10 participants provided positive feedback on the usability of the dashboard and expressed their satisfaction with how intuitive, easy to use, and helpful the tool has been. Their perception of the dashboard focused on 3 areas in particular: the usefulness of the dashboard in their practice (*Theme: Participants Found the Dashboard Helpful and Useful for Their Daily Tasks*); the intuitiveness of the design (*Theme: Participants Found the Dashboard Intuitive and Easy to Use*); and the usefulness of the data visualizations, in particular (*Theme: Participants Found the Data Visualizations Useful*). The hierarchy of the themes in this topic is shown in Figure 4.

#### Theme: Participants Found the Dashboard Helpful and Useful for Their Daily Tasks

Participants reported that the introduction of the dashboard had a positive impact on their ability to perform daily tasks. The responses frequently contrasted the qualities of our tool with those of the existing systems, highlighting the benefits of the dashboard and the change it brought on:

It’s a lot more difficult to see on the other systems, you have to log in to each individual patient and so having an overview like you do with the dashboard...It’s very helpful.

Nurse #2029

Presenting the data in a dashboard format was beneficial to the participants’ experience in the ward:

...the ventilators, the CIS, the blood results, blood gases, the tidal volumes from the ventilators, all that stuff is not always very easy to assimilate plus people’s infection status which you may not have necessarily gotten hold of, it’s all easier to get hold of in the dashboard format.

Consultant #2865

#### Theme: Participants Found the Dashboard Intuitive and Easy to Use

Overall, the user interface of the dashboard was received positively and frequently described as intuitive, clear, and easy to use. In their experience of familiarizing themselves with the dashboard, participants found its interface to be self-explanatory:

It was easy to use and it was very intuitive and it was quite self-explanatory really.

Consultant #2608

One participant implied that the dashboard was intuitive enough not to warrant any additional training materials:

So I did watch the videos and I have read the help page on the dashboard, but it’s pretty straightforward to use, to be honest.

Consultant #2885

#### Theme: Participants Found the Data Visualizations Useful

In their experience with various views of the data that included visualization, both in the table view (eg, trend indicators and color highlights) and bed view (eg, line charts), participants found the data visualizations to be particularly useful. The visualizations provided captured the attention of the participants more effectively than the other display formats:

...I think what they do is they draw your attention to things more rapidly...

Consultant #2313

Some participants found the utility of visually analyzing trends to apply to all patient types:

...the graphs where you can have the 24-hour view, the 48-hour view, the week you know since admission all that stuff, I find all that quite useful as a kind of trend view.

Consultant #2865

Other participants highlighted their usefulness for patients with large quantities of data, such as those with prolonged stays in the ICU:

...for those longer-term, complicated COVID patients that have been here for quite a long time, those graphical views are really useful.

Junior doctor #2462

### Topic: Dashboard Had a Positive Impact on the Delivery of Patient Care

#### Overview

**Figure 5:**
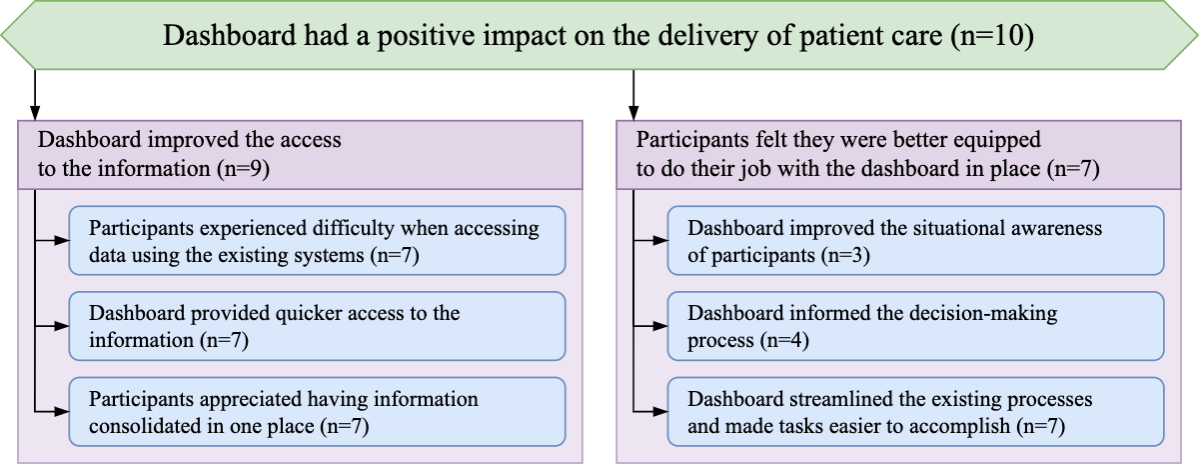
Hierarchy of the theme structure for the topic “Dashboard had a positive impact on the delivery of patient care.”.

In addition to the perceived usefulness of the dashboard, participants also reported on how the introduction of the tool impacted their delivery of patient care. To aggregate the themes that appeared throughout the interviews, 2 subtopics were generated, namely *Subtopic: Dashboard Improved the Access to the Information* and *Subtopic: Participants Felt They Were Better Equipped to do Their Job With the Dashboard in Place*. The hierarchy of the themes for this topic is shown in Figure 5.

#### Subtopic: Dashboard Improved the Access to the Information

##### Overview

The dashboard was introduced as a tool that would streamline access to the information and consolidate the most relevant data points in 1 place. Participants spoke extensively about the challenges associated with accessing the data using the existing systems (*Theme: Participants Experienced Difficulty When Accessing Data Using Existing Systems*), their experience of access to the information via the newly deployed dashboard (*Theme: Dashboard Provided Quicker Access to the Information*)*,* and frequently highlighted the benefit of having all relevant information available in 1 place (*Theme: Participants Appreciated Having Information Consolidated in 1 Place*).

##### Theme: Participants Experienced Difficulty When Accessing Data Using Existing Systems

To understand how access to information changed following the introduction of the dashboard, it is crucial to first present the experience of how the staff accessed the data using the existing systems. Participants reported challenges associated with access to information and the poor usability of the existing systems. They reported having to spend a lot of time navigating the existing systems in search of relevant information:

...it is all on [our CIS], but sometimes organised in a way that you have to do an awful lot of clicking.

Junior doctor #2462

Participants spoke about the large number of systems they needed to access in their daily jobs:

...one thing that we definitely struggle with is having too many systems.

Junior doctor #2462

Participants also spoke about the information being spread out across a variety of different systems:

...if you have a new admission that’s got links outwards to other care systems which can be used to gain further information on a patient, which is their past medical history on [our EPR] or their medications history on [our regional shared care record] or any other notifications that we might come up with on [our EPR]. [Our order-comms platform] as well is a useful link.

Junior doctor #2794

##### Theme: Dashboard Provided Quicker Access to the Information

Participants were able to access the information using the dashboard much quicker when compared with the existing systems:

...trying to find that out on [our CIS] is quite tricky, because you have to filter through every single patient, whereas because it’s displayed on a dashboard here, I can do it much quicker.

Consultant #2885

They also felt that our system was easier to navigate, which resulted in being able to access the desired data sooner:

I was sort of trying to use it as a way of getting a better overview of the patient more quickly, without having to click through all of the different bits.

Junior doctor #2462

##### Theme: Participants Appreciated Having Information Consolidated in 1 Place

The dashboard was used to access the information that was previously distributed across several other systems. To that extent, participants highlighted the benefits of having data from disparate sources consolidated in 1 place:

So I have used the dashboard a bit to try and get the information more in one place. But I have found it in some ways, it’s very helpful in that it’s, it’s sort of simpler because it’s just one line with all of the information.

Junior doctor #2462

Some participants also reflected on how the design of the dashboard and its focus on a specific problem improved their ability to assimilate information in comparison with the existing systems that presented all available data:

I think the point about the dashboard is it’s a much more concise amount of information...

Consultant #2608

#### Subtopic: Participants Felt They Were Better Equipped to do Their Job With the Dashboard in Place

##### Overview

In addition to the improvements in data access efficiency, the deployment of the dashboard aimed to enhance the quality of patient care and improve the experience of managing increased patient loads for participants. Participants reported numerous ways in which the dashboard improved their perception of the situation in the ward (*Theme: Dashboard Improved the Situational Awareness of Participants*), allowed them to make more informed decisions (*Theme: Dashboard Informed the Decision-Making Process*), and transformed existing methods of caring for patients (*Theme: Dashboard Streamlined the Existing Processes and Made Tasks Easier to Accomplish*).

##### Theme: Dashboard Improved the Situational Awareness of Participants

The dashboard delivered a snapshot of an entire ward, with disparate data sources aggregated in a single view. This allowed participants to get an overview of the situation in the ward, both when working in the ICU and remotely:

...where I found it useful at least, is before coming to do a week on the ICU, for example, it’s quite nice to have an overview of...how patients look, how the unit looks, how busy it is, how much COVID there is, how well or not the patients are doing and so for me to be able to have a snapshot view of knowing what I’m coming into without having to log into the CIS...is a useful thing to do.

Consultant #2865

By consolidating the relevant information in 1 place, the dashboard facilitated an ability to stratify patients based on priority and focus on the most critical tasks at hand:

...in a way a quick look at the dashboard is like okay those things are fine so now I can concentrate on what is actually going on with the lungs...

Consultant #2608

##### Theme: Dashboard Informed the Decision-Making Process

The introduction of the dashboard influenced the clinical decisions that participants made in the ward by providing a novel view of the data. Participants reported that the insight into the patient’s state delivered by the dashboard informed their decision-making:

[sorting patients by sequential organ failure assessment score has] proven really helpful to just understand that there’s a level of acuity and whether that’s getting better or worse, which sort of informs my decision making.

Consultant #2885

Being able to share the relevant data captured in a single view allowed for a more holistic insight, particularly during handovers. Presenting these data when discussing patient trajectories was highlighted as a useful tool:

...data that’s contained within the dashboard is a bit more objective data and with the hand over often between the two doctors is useful because you hear about things that aren’t necessarily in the notes but I think if you can supplement that with a data trend so that somebody who’s coming after you’ve looked after a patient for a week and see what progress you’ve made over the course of that week that might inform that conversation a little bit.

Consultant #2865

##### Theme: Dashboard Streamlined the Existing Processes and Made Tasks Easier to Accomplish

Facilitating easier access to the data resulted in efficiency improvements across different tasks tackled by the participants on a daily basis. These improvements suggested specific use cases for which the dashboard has proven to be especially useful. Some participants suggested using the dashboard for capacity planning:

But increasingly, it’s very useful for capacity planning. Because a number of the nuggets of information I need to supply...are quite readily available from it.

Consultant #2885

Other use cases were related to task management in the ward and involved using the dashboard for internal communication:

The things that the dashboard can display is the stuff that frankly anyone in intensive care can address and it’s there on a dashboard and it’s reliable.

Consultant #2608

Finally, the changes to their workflow stemming from the use of the dashboard proved useful to the participants and resulted in a more streamlined process:

I say anything that can streamline that workflow a bit and give you a sort of shortcut that helps you get to grips with somebody a bit quicker is useful and I’m very keen on that and I think that actually objective and sort of focused information is what I’m always looking for so anything that makes that a bit easier is of interest to me definitely.

Consultant #2865

### Topic: Dashboard Was Used in a Variety of Ways Across the Participants

#### Overview

**Figure 6:**
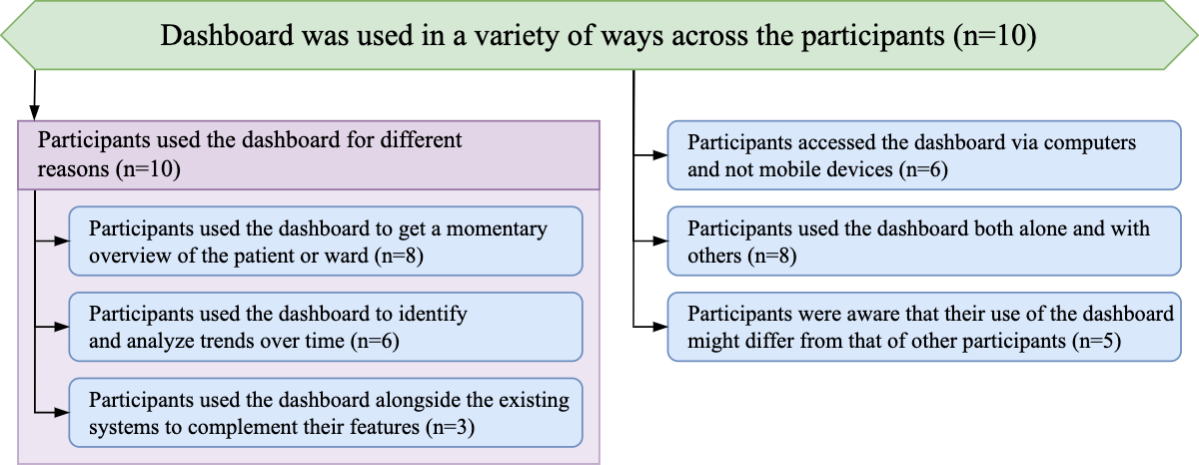
Hierarchy of the theme structure for the topic “Dashboard was used in a variety of ways across the participants.”.

A theme that prevailed across all interviews was the disparity in how the dashboard was being used by the participants. These differences included the reasons for use (*Subtopic: Participants Used the Dashboard for Different Reasons*), the different devices used to access the dashboard (*Theme: Participants Accessed the Dashboard via Computers and Not Mobile Devices*), and whether they accessed it together or alone (*Theme: Participants Used the Dashboard Both Alone and With Others*). The interviews also suggested that participants were aware of the differences between how they themselves use the dashboard and how other staff in the ICU can use it depending on their role (*Theme: Participants Were Aware That Their Use of the Dashboard Might Differ From That of Other Participants*). The hierarchy of the themes for this topic is shown in Figure 6.

#### Subtopic: Participants Used the Dashboard for Different Reasons

##### Overview

Our dashboard provided 3 views of the data: an overview with tiles presenting key parameters for each bed, a table aggregating information in greater detail, and a bed view that provided line charts for a single patient. Collectively, all participants reported using each of the 3 views and described how different aspects of the dashboard helped them use it in their practice. There were 3 prominent use cases that appeared in the interviews: the dashboard served as a snapshot of the situation in the ICU (*Theme: Participants Used the Dashboard to Get a Momentary Overview of the Patient or Ward*); it also provided a convenient way to spot and investigate change over time (*Theme: Participants Used the Dashboard to Identify and Analyze Trends Over Time*); and finally, it was used together with existing systems (*Theme: Participants Used the Dashboard Alongside Existing Systems to Complement Their Features*).

##### Theme: Participants Used the Dashboard to Get a Momentary Overview of the Patient or Ward

The dashboard was used to obtain a snapshot of the ward and enabled the participants to better understand the situation in the ICU:

I think the biggest change that it has given me at the moment is the ability to plan my day better and to understand an overview of our situation and I think it’s really, really useful for that.

Consultant #2885

It also allowed the users of our dashboard to access this overview without having to navigate the complex interfaces of the existing systems. In particular, this streamlined access was used as a shortcut to the information in time-critical scenarios:

[I used the dashboard] when doing the ward round [to give it] a quick glance or in a meeting [when I] haven’t got time to log into the whole [CIS] system to have an overview of where things are.

Consultant #2180

##### Theme: Participants Used the Dashboard to Identify and Analyze Trends Over Time

In addition to the momentary snapshot that the dashboard provided, participants reported using the data visualizations for a better understanding of how the situation in the ICU changes over time. Crucially, the dashboard allowed participants to understand the patient trajectories and assemble a care plan for the future:

[the dashboard allowed me to concentrate on] where are we compared to yesterday and where are we going and how are we going to progress this patient.

Consultant #2608

Visualizing the patient data using line charts also made it easier for participants to assimilate trends over larger spans of time:

...you could predict that someone was having a PE by the changes in their AA-gradient or something like that, some things that might not be obvious to the eye, but by calculating trends [they] might become.

Consultant #2357

##### Theme: Participants Used the Dashboard Alongside Existing Systems to Complement Their Features

In addition to the stand-alone use of our tool, the participants also used the dashboard in conjunction with the existing systems. The dashboard was used as an extension when performing data entry tasks within the electronic patient record:

...what I tried to use it for is when I’m updating the list, it would be good to like look at patients and basically be able to get a really quick view of the things that we look at, which was the ABCD kind of assessment, and then the systems-based assessment...

Junior doctor #2462

Significantly, this joint use of the systems stemmed from the ability to obtain the required information from the dashboard more efficiently, despite already using the existing systems:

It was quite useful to be able to see everything for updating [our EPR] before hand over because it was a quick way to get information.

Junior doctor #2638

##### Theme: Participants Accessed the Dashboard via Computers and Not Mobile Devices

The initial requirements elicited for the tool specified the need for the dashboard to be available on mobile devices, such as the tablets available at the trust [23]. As such, the dashboard was developed as a PWA to enable access across a wide variety of devices, including mobile devices. Despite this, participants reported dashboard use primarily on their desktop computers or laptops and not on mobile devices:

I’d say I never had it running on an iPad, so not when walking around on the ward round but on the desktop, or on the portable computers. On the laptops I have used it.

Consultant #2357

The nature of the job role and its associated responsibilities also influenced the choice of desktop computers to access the dashboard:

I tend to use desktop computers as the consultant because I suppose I tend to anchor myself at desk and then sort of do a slightly scattergun approach to try and get around the patients.

Consultant #2885

Finally, participants also attributed their preference for not using mobile devices to the intrinsic limitations of mobile devices:

...making sure that I can use a bigger screen and someone doesn’t keep turning it off, or the battery goes off. So I tend to use a desktop by a nursing station.

Consultant #2885

##### Theme: Participants Used the Dashboard Both Alone and With Others

Participants used our dashboard to assist with a variety of tasks. Among those, some tasks such as patient handover and performing ward rounds featured frequently in the interviews. When asked about the use of the dashboard during these tasks, participants reported having used the dashboard together with others:

[I used it] on ward rounds and stuff, a little bit with other people.

Nurse #2029

The ability to use the dashboard together helped the participants in communicating with their peers and enhanced the communication between staff:

The handover often between the two doctors is useful because you hear about things that aren’t necessarily in the notes but I think if you can supplement that with a data trend.

Consultant #2865

In addition to these tasks, the dashboard also served as a personal tool for gaining insight into the current situation in the ward. In particular, at the times of limited resources in the ICU such as night shifts, participants turned to our dashboard to supplement their understanding of the current condition of the patients:

No, I definitely just use it on my own as well, like, particularly on night shifts when you kind of try and get an idea of how patients are doing overnight.

Junior doctor #2462

##### Theme: Participants Were Aware That Their Use of the Dashboard Might Differ From That of Other Participants

During the interviews, participants shared their opinions on how their peers might use the dashboard and compared it with how they themselves use it in their practice. This awareness of the disparities between the responsibilities prompted them to reflect on the usefulness of different features in the context of their job roles within the ICU:

But it’s certainly in the context of the way that I’m using it. And I’m sure if I was looking after long-term patients, I’d be very interested in those trends.

Consultant #2885

They also suggested providing different views for different stakeholders to better cater to those responsibilities:

Yeah, you can probably have a different setup for different people...I guess, for me, it’d be like respiratory rate FiO2, PEEP, but then I know consultants also use things like the minute volume and things like that.

Junior doctor #2462

The dashboard was also suggested as a tool that might help ease the new staff members into the ICU workflow by providing a more accessible interface:

...for somebody who comes in as a brand new trainee I think it’s a slightly overwhelming system when you’re starting, there’s just a huge amount of information coming at you and it’s probably really, really hard to synthesise and so to synthesise that with something more visual or graphics to somebody who isn’t used to all this coming at them would be easier.

Consultant #2180

### Topic: Participants Experienced Barriers Integrating the Dashboard Into Their Workflow

#### Overview

**Figure 7:**
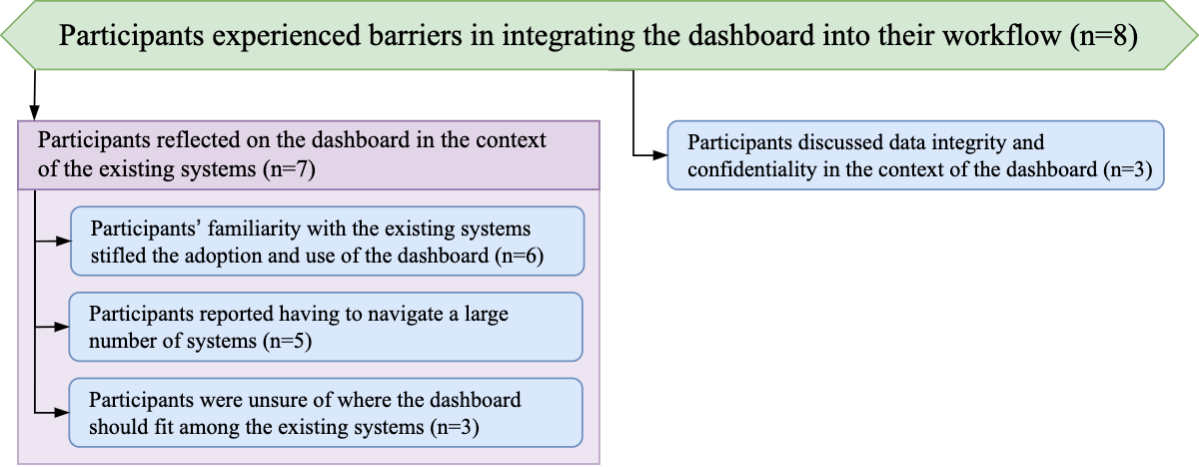
Hierarchy of the theme structure for the topic “Participants experienced barriers integrating the dashboard into their workflow.”.

The overall reception of the dashboard was positive, both in terms of satisfaction with its usability and the way in which it transformed the delivery of patient care. Despite that, during the deployment in the ICU, some participants experienced barriers that prevented them from fully embedding this new tool within their workflow. A theme that appeared frequently in the interviews was the purpose and use of the dashboard in the environment, which was already saturated with a number of digital systems in place (*Subtopic: Participants Reflected on the Dashboard in the Context of Existing Systems*). Participants also voiced their opinions on how the deployment of the dashboard affected the issues of information security (*Theme: Participants Discussed Data Integrity and Confidentiality in the Context of the Dashboard*). The hierarchy of the themes for this topic is shown in Figure 7.

#### Subtopic: Participants Reflected on the Dashboard in the Context of Existing Systems

##### Overview

The ICU is an environment that is already saturated with a variety of information systems, each frequently serving its own, unique purpose. Integration of our tool into this landscape, particularly during the pandemic, proved to be challenging. Specifically, participants emphasized that during the increased workload created by the COVID-19 pandemic, they frequently resorted to using systems they were already familiar with (*Theme: Participants’ Familiarity With the Existing Systems Stifled the Adoption and Use of the Dashboard*), they also highlighted the numerous systems they already needed to navigate (*Theme: Participants Reported Having to Navigate a Large Number of Systems*), and they expressed uncertainty of where the dashboard belonged among those systems (*Theme: Participants Were Unsure of Where the Dashboard Should Fit Among Existing Systems*).

##### Theme: Participants’ Familiarity With the Existing Systems Stifled the Adoption and Use of the Dashboard

The dashboard was deployed in the ICU during the COVID-19 pandemic. During this time of uncertainty and constantly evolving situation, the participants worked under extreme conditions and with severely limited resources. As a result, some participants found it difficult to integrate the dashboard into their workflow and reverted to using the systems they were already familiar with despite the lack of usability barriers to the existing system:

I guess in terms of limitations and barriers to it, the past several shifts I guess have been clinical busyness during the day time and overnight, you kind of just want to use something that is tried and tested, something that is a familiar system. That’s not to say one system is better or worse...[our CIS] is what I’ve been using for the past several months so it’s what I’ve become accustomed to. I have tried the dashboard in itself and the user interface I’d say is quite clear to use and useful...

Junior doctor #2794

The participants also suggested that integrating the dashboard into their workflow before the pandemic could have alleviated the issue of unfamiliarity:

I’ve found that when we were overwhelmed with COVID patients, which probably one would have been the most useful—that’s the time when you tended to sort of go back to basics and almost leaving some of these tools behind, which is a bit of a shame. But I think that if it was embedded fully before, that would have been better...

Consultant #2313

Although our tool has often resulted in a more streamlined process overall, embedding it into participants’ practice demanded changes to how they currently worked:

I’ve had to adapt my way of working in order to integrate it into my workflow.

Consultant #2865

##### Theme: Participants Reported Having to Navigate a Large Number of Systems

Owing to the multifaceted nature of the work in the ICU, which relies heavily on the collaboration between different departments, there are a vast number of digital systems that participants have to navigate on a daily basis. This results in the information being spread out across multiple places and makes effective access to that information challenging:

I don’t want to have to go into two or three different systems to have to see what’s going on I want to go to one place and have all the information given to me and then I can drill into when I need to.

Consultant #2180

The need of already having to navigate a large number of systems had a negative impact on the adoption of the dashboard and prevented some participants from effectively embedding the dashboard within their workflow. In the interviews, participants emphasized the need to design new tools with an integration strategy in mind:

I guess because it’s not in my workflow yet so I have to make an effort to go out and look at it, it’s another thing if you like and the key is to really try and get it integrated so that I could see it.

Consultant #2180

##### Theme: Participants Were Unsure of Where the Dashboard Should Fit Among Existing Systems

Owing to the variety of systems present in the ICU, participants frequently turn to different tools for different tasks. This suggests that each tool serves a unique purpose that distinguishes it from the other systems. Although the role of the dashboard focused on streamlining the access to the information that participants could already access, albeit through a more challenging and laborious process, some participants expressed uncertainty regarding the unique purpose of the dashboard:

I don’t quite know how it fits in with the systems that we already have.

Junior doctor #2462

One of the suggestions provided by participants as means to improve the adoption of the dashboard was to extend its set of features beyond those of the existing systems:

...it’s displaying the same things that are displayed in [our CIS], predominantly, just graphically rather than numerically...I think it would be useful to have it put towards specific tasks that [our CIS] doesn’t do well.

Consultant #2357

Participants also suggested that having an internal “champion” to facilitate the integration of the dashboard would improve its adoption within the ICU:

I suppose the only thing with the dashboard is like sometimes people get a bit of dashboard fatigue it’s like it’s been there and no one ever updates it or looks at it and then it just becomes part of the furniture so I think it probably needs champions in each area like nurses, in particular, to encourage its use.

Consultant #2608

##### Theme: Participants Discussed Data Integrity and Confidentiality in the Context of the Dashboard

The dashboard was made available to all the staff members of the ICU through an internal network URL. To access that address, participants had to first sign in to the devices on the network; however, once connected, the dashboard did not require further authorization. This removed the potential barrier to use by making the process of accessing the data quicker but prompted participants to reflect on the data confidentiality aspect of the dashboard:

...I think it was just a link and I don’t know if there are plans to have a login to access the

dashboard] or whether it can only be done via the intranet or trust computers...[Junior doctor #2794

Participants also mentioned the issue of data integrity, in which the information displayed across the different systems may differ:

I was quite worried about data integrity - so “is it truly representing what’s going on?” - but I’m very reassured that every time I use it seems to be [reflecting] what is happening.

Consultant #2885

Despite these potential concerns, participants felt that the dashboard presented no issues that could affect patient care:

No concerns from either a patient safety point of view or a confidentiality point of view.

Junior doctor #2794

## Discussion

### Principal Findings

#### Overview

This study sought to evaluate the dashboard built in response to the global COVID-19 pandemic. By introducing the dashboard, we aimed to alleviate the challenges associated with the high ICU census caused by the pandemic. Consequently, our tool was designed to improve access to information and make it easier to assess and understand the current situation in the ward.

#### Improved Patient Care

Zhuang et al [33] suggest that in the context of dashboards within the health care setting, “providing users with a positive experience is the ultimate goal of developing any type of information system.” In our interviews with the staff who used the dashboard clinically during the pandemic, the participants reported satisfaction with the tool and the tangible benefits it brought to their workloads surrounding patient care. The intuitive nature of the user interface and the ability to consolidate disparate data sources from existing systems had the greatest impact on improving access to information. These findings are in line with the existing evidence of dashboard efficacy on patient care [4,10] and highlight the role of information accessibility in streamlining the clinical processes.

The current body of knowledge further suggests that dashboards have the potential to influence situational awareness [34] and increase the efficiency of workloads that rely on effective access to information [8,9]. This is reflected in the evidence from our study, in which participants reported that better access to information and the ability to assimilate it quickly made them more aware of the situation in the ward, leading to a faster and more effective triage process.

The previously impossible overview of the key data points spanning patients across the entire ward generated new insights that informed the decision-making process and provided participants with a more holistic view of the data. In addition, the use of our dashboard provided efficiency improvements to the existing collaborative tasks such as patient handover and served as a robust basis for communication between the staff. The current literature on the effects of dashboards on team workflows and patient handoff supports these findings [35].

#### Modes of Use

The reported use of the dashboard varied substantially among the participants both in terms of mode of use and the underlying purpose. Although prior research suggested that participants wanted a mobile dashboard (ie, accessible via portable devices such as tablets) [23], participants reported using the dashboard primarily on desktop computers and laptops. Implementing the application that targeted mobile devices and adapting the features to work within their limitations had a significant impact on the design and development of the software itself. The need to incorporate features such as PWA installability, support for touch screen–specific gestures, and an interface that fits on a smaller display size further prolonged the development time and impacted the usability of the dashboard.

Despite this, the participants reported extensively on the ways in which they used the dashboard in clinical practice. These included coordinating activities that involved multiple staff, such as patient handover, in which the dashboard served as a ground truth and tool for communicating with others, or ward rounds during which participants used the dashboard to quickly assess and stratify patients. Furthermore, participants also used the dashboard on their own to improve their situational awareness and gain a better understanding of the state of the ICU, particularly when the resources available to them were more limited (eg, night shifts) or in time-critical scenarios when quick access to information was paramount (eg, ward rounds). Within these use cases, 2 prominent modes of use were observed. First, the participants used the tool to obtain a momentary snapshot of the ward, providing the most recent and relevant information across the entire ward with ease. Second, the tool also enabled participants to view and analyze trends and patterns over time through its use of data visualizations. Finally, the dashboard was also used in conjunction with the existing systems and allowed the participants to supplement the features of those systems with the ability to rapidly assimilate data across a variety of patients, specifically during the data entry–related tasks.

#### Integration Barriers

One of the key findings of our study emphasizes the importance of integrating the new digital tools into health care settings and, in particular, the existing workflows of the staff. Although the introduction of the dashboard within the ICU was met with an overall positive reception and resulted in improvements to the efficiency of existing processes, participants highlighted barriers that inhibited the adoption of our tool into their daily workflows. Notably, these barriers were largely related to the participants’ familiarity with the existing systems and the workflows previously established while working in the ICU.

The COVID-19 pandemic made the already challenging task of managing patients needing critical care even more difficult. The sudden influx of patients, which drastically increased the staff-to-patient ratio, and the constantly evolving guidance for the treatment process resulted in immense pressure on the clinical staff. In this time of need, participants turned to the tools they were already familiar with in an attempt to decrease the cognitive load they experienced; this further stifled the adoption of the newly developed dashboard. Participants also suggested that they would have used the dashboard more if it was already integrated into their workflow at the time of the pandemic.

The staff reported feeling overwhelmed by the wide variety of different systems they were required to navigate on a daily basis to collate the relevant information. Although the dashboard aimed to consolidate the information from variety of systems into 1 tool and reportedly assimilated the issue of having to navigate multiple systems to find the necessary information, the introduction of yet another tool into the workflow contributed to the number of systems available to the staff. This difficulty in managing a growing number of digital tools within the critical care setting, also referred to as “dashboard fatigue” by one of the participants, prompted reflection on the unique purpose of the dashboard in the landscape of digital systems present within the ICU. Participants suggested nominating a person to champion the tool to improve its adoption and suggested that the dashboard should focus on the aspects of the existing systems that are not being used effectively.

### User Acceptance

The qualitative evidence captured during this study suggests that participants found the dashboard both useful and easy to use. In the context of TAM, the perceived usefulness and ease of use indicate that users are willing to adopt and integrate the technology, both because they perceive it as valuable and relevant to their needs and because it reduces the effort required to learn the software [29,36]. This is reflected in our findings. However, it is worth noting that the adoption of our dashboard was affected by the external influence of the global COVID-19 pandemic, which imposed additional complications on the acceptance and integration of the dashboard into practice. Failing to incorporate the external variables while assessing the technology acceptance is one of the frequently cited limitations of TAM [37,38].

### Limitations

Both the study and the development of the dashboard took place during the COVID-19 pandemic. This imposed limitations on participant recruitment and the structure of the study. As at the time of the pandemic, the priority in the ICU focused heavily on ensuring clinician and patient safety, the available pool of potential participants willing to participate in the research was significantly reduced. The increased workload and challenging work conditions made it much more difficult to find time for testing the new dashboard and participating in the interviews than it would have been under normal circumstances. This resulted in the data collection process spanning 99 days across all participants, which could have had an impact on the provided responses (eg, some participants would have used the dashboard for longer than others at the time of the interview). Both the uptake and long-term adoption of the dashboard were also affected by the tendency of the participants to use the systems they were already familiar with to further reduce their cognitive burden. The dashboard was built to support mobile devices such as tablets, which contrasted with the actual use patterns of the participants who primarily used it on desktop computers and laptops. The software was also designed to accommodate the expected patient loads of the NHS Nightingale Hospitals, which would have differed significantly from those of the UHBW and would have likely resulted in different modes of use. Finally, the experiential nature of the results suggests that further evaluation using quantitative methods is necessary.

### Conclusions

This study outlines the process of the design, development, and deployment of a bespoke ICU dashboard during the COVID-19 pandemic based on prior work that focused on capturing the end users’ requirements. It introduces the evaluation of the dashboard’s utility and informs the future efforts of building dashboards within the critical care setting. To that extent, the study presents the findings from the thematic analysis conducted on the transcripts of the semistructured interviews with participants.

The analysis highlighted participants’ satisfaction with the dashboard and the positive impact it had on patient care. It also illustrated the different modes of use present among the participants, provided evidence on the barriers to integration encountered during the deployment, and participants’ suggestions to improve the adoption.

We stated and discussed the limitations of our study and addressed them by proposing future directions for research in this area. Despite these limitations and the challenges in integrating the dashboard within the workflow of clinicians during the pandemic, participants reported a significant impact on their experience of patient care delivery. This suggests a critical need to further investigate the use of dashboards in the critical care setting and explore how these promising tools could continue to improve modern clinical practice.

### Future Work

Three key limiting factors of this study should be addressed in future studies. First, the evaluation focused on the self-reported and subjective measures of the dashboard’s utility in the ward. To fully understand the impact of the dashboard on patient care, more objective (eg, outcome based) measures should be used to supplement this qualitative analysis. To that extent, we suggest a study design that encompasses the evaluation of quantifiable performance metrics, such as those reported by Bourdeaux et al [10].

Second, the dashboard addressed the problem of information access and more effective delivery of insights stemming from existing data. Although this allowed direct measurement of the impact of a dashboard format, research on the use of dashboards for delivering processed data (eg, analytics or machine learning predictions) could further inform how the use of dashboards influences clinical practice.

Third, there are significant infrastructural challenges associated with capturing objective data on the influence of interventions such as dashboards. Work focusing on improving the availability of the latent data for the purpose of evaluation and research [39] should be continued to enable better assessment of future interventions.

## Appendix

Multimedia Appendix 1 Interview guide used during the semistructured interviews.

## Abbreviations

ICU: intensive care unit
NHS: National Health Service
PWA: progressive web application
TAM: Technology Acceptance Model
UHBW: University Hospitals Bristol and Weston National Health Service Foundation Trust
